# The effect of stoichiometry on the structural, thermal and electronic properties of thermally decomposed nickel oxide†

**DOI:** 10.1039/c8ra00157j

**Published:** 2018-02-06

**Authors:** P. Dubey, Netram Kaurav, Rupesh S. Devan, G. S. Okram, Y. K. Kuo

**Affiliations:** Department of Physics, Government Holkar Science College A. B. Road Indore 452001 MP India netramkaurav@yahoo.co.uk; Discipline of Metallurgy Engineering and Materials Science, Indian Institute of Technology Indore Khandwa Road, Simrol Indore 453552 India; UGC-DAE Consortium for Scientific Research, University Campus Khandwa Road Indore 452001 MP India; Department of Physics, National Dong Hwa University Hualien 97401 Taiwan

## Abstract

A thermal decomposition route with different sintering temperatures was employed to prepare non-stoichiometric nickel oxide (Ni_1−*δ*_O) from Ni(NO_3_)_2_·6H_2_O as a precursor. The non-stoichiometry of samples was then studied chemically by iodometric titration, wherein the concentration of Ni^3+^ determined by chemical analysis, which is increasing with increasing excess of oxygen or reducing the sintering temperature from the stoichiometric NiO; it decreases as sintering temperature increases. These results were corroborated by the excess oxygen obtained from the thermo-gravimetric analysis (TGA). X-ray diffraction (XRD) and Fourier transformed infrared (FTIR) techniques indicate the crystalline nature, Ni–O bond vibrations and cubic structural phase of Ni_1−*δ*_O. The change in oxidation state of nickel from Ni^3+^ to Ni^2+^ were seen in the X-ray photoelectron spectroscopy (XPS) analysis and found to be completely saturated in Ni^2+^ as the sintering temperature reaches 700 °C. This analysis accounts for the implication of non-stoichiometric on the magnetization data, which indicate a shift in antiferromagnetic ordering temperature (*T*_N_) due to associated increased magnetic disorder. A sharp transition in the specific heat capacity at *T*_N_ and a shift towards lower temperature are also evidenced with respect to the non-stoichiometry of the system.

## Abstract 2

A thermal decomposition route with different sintering temperatures was employed to prepare non-stoichiometric nickel oxide (Ni_1−*δ*_O) from Ni(NO_3_)_2_·6H_2_O as a precursor.

## Graphical abstract

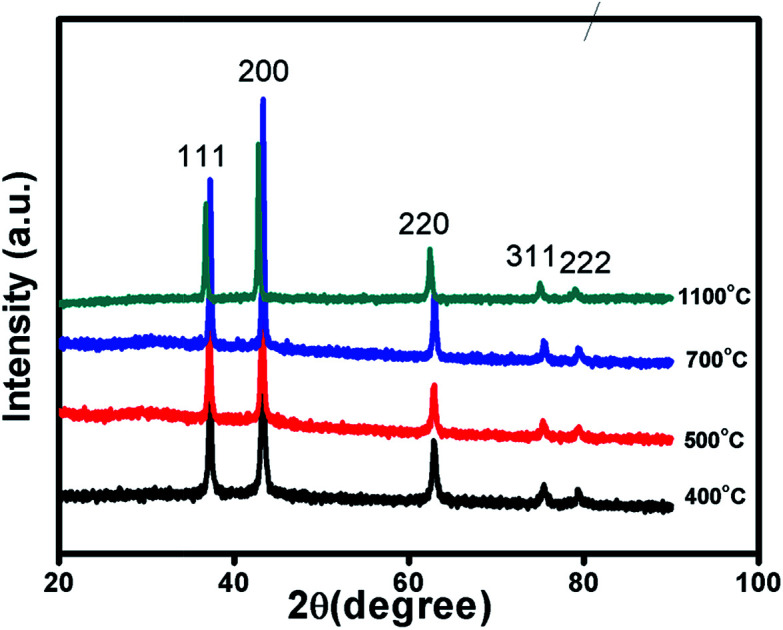

## 1. Introduction

As an antiferromagnetic^1^ and Mott–Hubbard insulator,^2^ stoichiometric nickel oxide (NiO) has been the most exhaustively investigated transition metal oxide. It has attracted considerable interest as a relatively low-cost, low-toxicity, and environmentally-friendly material. Such qualities make it an attractive material that is useful in a range of applications, such as transparent conductive film,^3^ chemical sensors^4^ and resistive random access memory.^5^ In addition, it is also a well-studied material as a positive electrode in batteries^6^ and in quantum dot light emitting devices as a hole transport layer.^7^ The performance of these devices depends on conditions of preparations. For example, on changing these conditions, NiO of different stoichiometry (Ni_1−*δ*_O) can be obtained, in which composition ratios between nickel and oxygen are not exactly 1 : 1. Because of excess oxygen and vacancies on Ni site, nickel oxide thus becomes a p-type semiconductor.^8^ Thermodynamically, it is found that this nickel vacancy is the most dominant point defect present in the system.^9^ Various characteristics of the nickel oxide such as electrical,^10^ optical^11^ and thermal^12^ properties depend strongly on its stoichiometry. The changes in physical properties are thought to be attributed to the oxidation state of nickel which changes with oxygen concentration, which eventually produces cation vacant nickel oxide. An excess of oxygen decreases with an increase in sintering temperature of the nickel salt. Densities of nickel oxide and activation energy of electrical conductivity decrease with an increase in the excess of oxygen whereas the lattice parameter do not vary.^13^ Isothermal change in electrical conductivity of nickel oxide shows that the decrease in vacancies may be attributed to diffusion of vacancies to the crystal surface.^14^

Depending on the conditions of preparation, the sintering temperature, in particular, NiO samples of various surface areas, color, and degree of non-stoichiometry can be prepared. Nickel oxide can easily be prepared *via* several methods, including chemical route, evaporation,^7^ sputtering,^15,16^ chemical deposition,^17–19^ oxidation of nickel,^20^ sol–gel method^21^ and thermal decomposition.^22^ In particular, thermal decomposition method is a broad category for its powder preparation that impacts on other techniques and is an important method in its own, because it is a simple, low-cost and fast endothermic process. Therefore, the present compound NiO provides a unique example of a non-stoichiometric compound. In the present study, we have investigated the effect of oxygen concentration in nickel oxide prepared by the thermal decomposition method. Excess oxygen was estimated by iodometric titration, which was corroborated by thermo-gravimetric analysis (TGA). It is found that the amount of excess oxygen or oxidation state of nickel in nickel oxide is closely related to synthesis temperature. Fourier transforms infrared (FTIR) spectroscopy is used to analyze the bonding of oxygen with metal ions as well as stoichiometry of the prepared samples. Using X-ray Photoelectron Spectroscopy (XPS) as a surface analytical method, binding information, chemical nature and valence states with compositional changes in the samples were investigated. Temperature dependence of magnetic susceptibility (*χ*) and heat capacity (*C*_p_) was then studied to analyze the effect of non-stoichiometry on transition temperature in Ni_1−*δ*_O samples.

## 2. Experimental

### 2.1 Synthetic of non-stoichiometric nickel oxide

Non-stoichiometric nickel oxide was obtained by thermal decomposition of nickel nitrate hexahydrate.^23,24^ Typically, about 5 g Ni(NO_3_)_2_·6H_2_O was decomposed thermally in open air for 3 hours at 400 °C to produce nickel oxide sample with a particular content of oxygen (Ni_1−*δ*_O). The product thus obtained was pure, as no catalyst is required in this method. The precursor breaks down in two or more products in the process in which the gaseous parts are escaped from the system freely. The kinetics of this process is such that it reveals the minimum time–temperature condition necessary for the decomposition. This sample was denoted as NiO400. Similarly, seven other samples were prepared at 500 °C, 600 °C, 700 °C, 800 °C, 900 °C, 1000 °C and 1100 °C. They were denoted NiO500, NiO600, NiO700, NiO800, NiO900, NiO1000 and NiO1100, respectively.

The Bruker D8 Advance X-ray diffractometer with Cu Kα radiation (0.154 nm) in the angle range 10–90° was used for the laboratory method of XRD measurements of the samples in powder form. X-rays were detected using a fast counting detector based on Silicon Strip Technology (Bruker Lynx Eye detector). To determine oxygen content, iodometric (redox) titration was used with standardized sodium thiosulfate and potassium iodide solution with a starch solution as an end-point indicator. Thermo-gravimetric analysis (TGA) was used to further corroborate the non-stoichiometry in these samples. The TGA system with the top of the line METTLER TOLEDO ultra-micro balance with unique built-in calibration weights ensures an accuracy of 0.1 μg.

In the Fourier transform infrared (FTIR) transmission measurements, a few micrograms of each sample were added to a fixed quantity of pure KBr and ground thoroughly. Then, a pellet of the uniform mixture was made for each sample. FTIR spectrum of a pure KBr pellet was subtracted from the FTIR data of each sample to obtain the corresponding FTIR spectra. X-ray photoelectron spectroscopy (XPS) measurements were done on a PHI 5600 CI (Physical Electronics) spectrometer using Al Kα non-monochromatic X-ray excitation at 350 W power, an analysis area of 0.8 mm in diameter and pass energy of 200 eV for electron analysis. The experimental error was below 0.1 eV while the spectrometer resolution was better than 1 eV. These data were used for identifying the compositions of Ni_1−*δ*_O samples. Magnetization measurements were done on VSM-SQUID (Quantum Design) with a maximum of 7 tesla field. Specific heat is obtained with modulated differential scanning calorimeter MDSC-2910 (TA Instruments).

## 3. Results and discussion

### 3.1 X-ray diffraction analysis

**Fig. 1:**
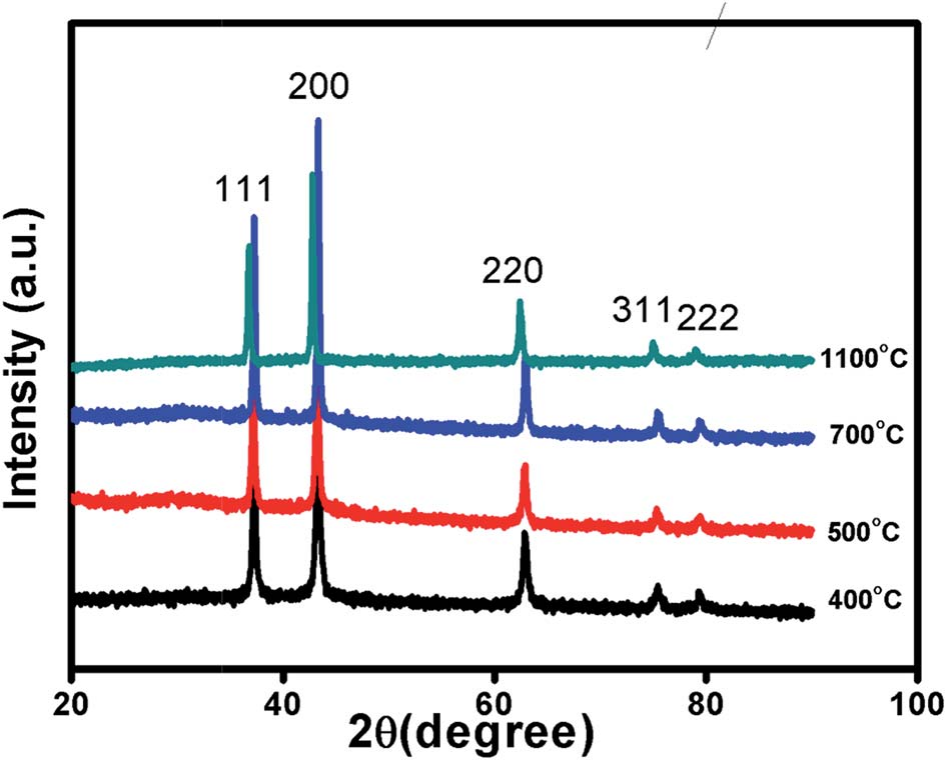
Characterization of sample. X-ray diffraction pattern of non-stoichiometric Ni_1−*δ*_O samples sintered at different temperature as indicated.

**Fig. 2:**
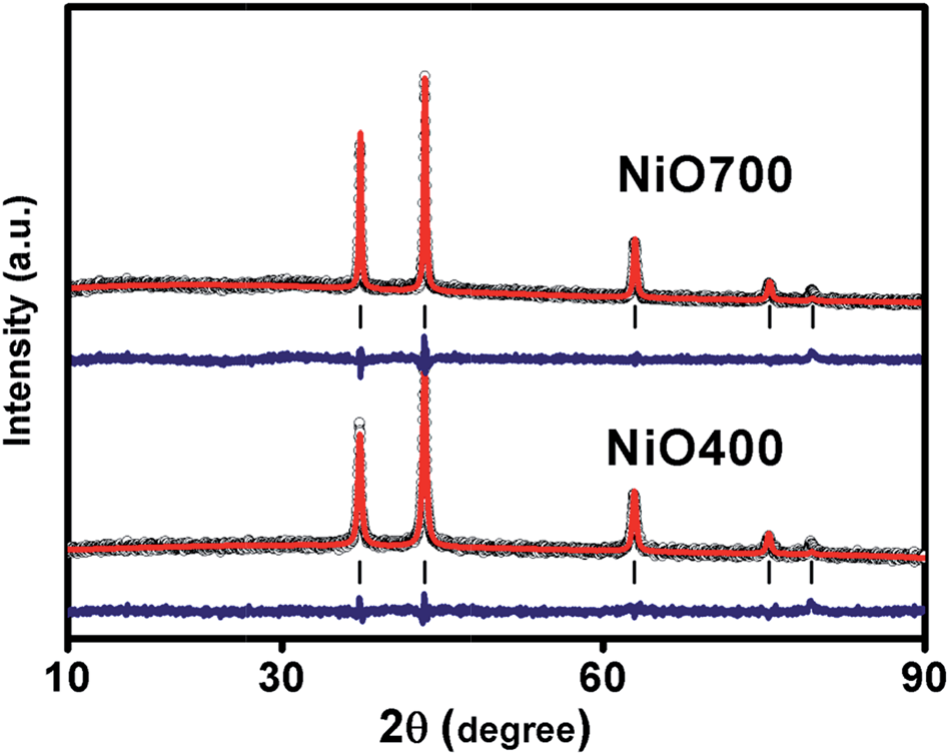
Shows the graphic results of the representative Rietveld refinement fitting of XRD data for NiO400 and NiO700. Experimental data (open circles), with Rietveld fitted curve (continuous red), vertical lines indicating peak positions and difference.

Fig. 1 and S1 in ESI† shows XRD patterns of Ni_1−*δ*_O samples, NiO400, NiO500, NiO700, NiO1100 and NiO600, NiO800, NiO900, NiO1000. XRD peaks match well with the standard XRD of NiO (JCPDS 47-1049) with no other impurity peaks, showing that these samples were of a single phase in nature in each of them. XRD patterns were profile-refined using the Full-Prof software package [http://www-llb.cea.fr/fullweb/]. Fig. 2 gives the representative Rietveld profile fit for NiO400 and NiO700 samples along with different patterns obtained by using *Fm*3*m* (225) space group. In overall, the XRD analysis shows that these samples remain single phase, *i.e.*, NiO face-centre-cubic (fcc) phase. The cell parameters of all samples listed in Table 1. Interestingly, the unit cell volume decreases as the excess oxygen of the sample decreases XRD patterns show diffraction peaks associated only to NiO, indicating that nickel–nitrate hexahydrate has been transformed mostly into NiO. However, some traces of organic compounds may still be present in samples prepared below 700 °C as discussed in the later section.

**Table 1:** Rietveld refinement parameters of nonstoichiometric nickel oxide samples

Samples	*a* (Å)	Volume (Å^3^)	*R*_f_ factor	Breggs, *R*-factor	*R*_p_	*R*_wp_	*R*_e_	*χ* ^2^
NiO400	4.1764 (±0.000934)	72.8460 (±0.028)	7.15	3.94	24	16.7	14.9	1.261
NiO500	4.1745 (±0.000)	72.7467 (±0.000)	8.01	4.33	29.6	19.6	16.7	1.381
NiO600	4.1738 (±0.00049)	72.7101 (±0.014)	8.10	4.15	24.6	16.1	14.8	1.191
NiO700	4.1732 (±0.000)	72.6787 (±0.000)	8.14	4.51	22	15.8	13.3	1.416
NiO800	4.1728 (±0.000)	72.6579 (±0.000)	8.46	8.58	27	18.4	15.6	1.397
NiO900	4.1721 (±0.000521)	72.6213 (±0.016)	12.0	12.0	39.5	26.9	17.0	1.157
NiO1000	4.1718 (±0.000)	72.6056 (±0.000)	19.7	29.4	44.7	33.3	17.5	1.16
NiO1100	4.1711 (±0.000321)	72.5691 (±0.011)	20.4	31.7	56.3	37.4	22.3	1.06

### 3.2 Excess oxygen determination

The changing of nickel ions oxidation state can be realized either by changing sintering temperature^25^ or the composition of a sample by diluting foreign atoms.^26^ In general, one expects an excess of oxygen in Ni_1−*δ*_O samples. To determine the content of oxygen in the presently studied Ni_1−*δ*_O samples, iodometric titrations were carried out using sodium thiosulfate (2.023 × 10^−3^ mol^−1^) as a titrant. Samples (*ca.* 0.025 g) were dissolved in a solution of KI and HCl (10.0 ml; *ca.* 0.1 mol^−1^ KI, *ca.* 0.1 mol^−1^ HCl). Resulting solutions were diluted to 25 ml and titrated immediately against thiosulfate solution. The starch indicator was added prior to the end-point being reached. The excess oxygen in NiO samples prepared at different temperatures is shown in Table 1. It was found that the compositional ratio of O/Ni is larger than 1 for samples prepared below 700 °C and it is nearly 1 for samples prepared above 700 °C.

**Fig. 3:**
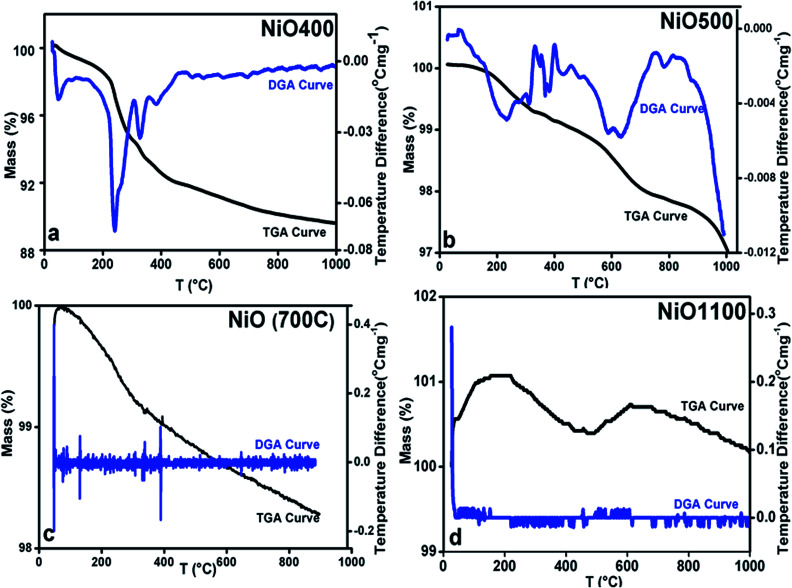
TGA curves of non-stoichiometric Ni_1−*δ*_O samples for (a) NiO400, (b) NiO500, (c) NiO700 and (d) NiO1100; heating rate of 5 °C per min was maintained in all.

This finding suggests that the nickel oxide prepared by heating the precursor below 700 °C has an excess of oxygen and hence the oxidation state of nickel changes with a change in sintering temperature. By iodometric titration, the amount of Ni^3+^ is also determined. Here, the result is expressed in percent atoms of oxygen in excess, by considering that two Ni^3+^ ions correspond to three ions of O^2−^. In order to further estimation of excess oxygen, TGA was performed in the inert atmosphere. In general, TGA is a technique that measures the change in weight of a sample as it is heated, cooled or held at constant temperature, which eventually characterizes materials with regard to their compositions. In the present investigation, the weight of the non-stoichiometric oxygen is obtained directly by obtaining the weight loss. The thermogravimetric (TG) curve and its differential curve (DGA curve-derivatographic analysis) are shown in Fig. 3(a) for NiO400 sample. It is evident from this figure that a very sharp peak is present between 200 and 300 °C on the DGA curve, which is a measure of the amount of excess oxygen released in the heating process. The corresponding weight loss reflects desorption of oxygen in the non-stoichiometric sample. On the other hand, the peak below 100 °C is due to the evaporation of the physically bound water in the sample and does not contribute to the estimation of excess oxygen. As shown in Fig. 3(b–d) by TG and DGA curves, it is noted that for NiO500 sample, the reduction of mass with temperatures decreases and becomes negligible for the sample prepared above 700 °C. The weight percentage of excess oxygen is tabulated in Table 2 from above TG curves. It can be seen from the Table 2 that the estimation of excess oxygen in these non-stoichiometric samples by two different methods corroborates with each other. In order to further explore, the essential feature of excess oxygen in these non-stoichiometric samples, we have prepared another NiO400 sample in the presence of oxygen flow during heat treatment. The TG curve and its DGA are shown in Fig. 3(a) and S2 in ESI,† respectively. Both the curves are nearly identical; however, the weight percentage loss is slightly less as compared to pristine NiO400 sample. The difference in loss of weight percentage is calculated and values are reported in Table 1. This might be due to the differences in the deficiency of nickel in NiO400 samples with and without oxygen annealing during heat treatment. Thus, heating the Ni_1−*δ*_O at higher temperatures heals the defects and leads to the corresponding atomic rearrangement. Thus, highly defective nickel oxide at lower temperature approaches stoichiometric nickel oxide as the heating temperature increases, presumably, due to change in oxidation state of nickel as the sintering temperature changes.

**Table 2:** Excess oxygen calculated from iodometric titration and TGA

Samples	% of excess oxygen in iodometric titration	% of excess oxygen in thermogravimetric analysis
NiO400	39.51	0.388
NiO500	39.46	0.388
NiO600	39.40	0.388
NiO700	39.38	0.388
NiO800	39.40	0.388
NiO900	39.41	0.388
NiO1000	39.57	0.388
NiO1100	39.41	0.388

### 3.3 Spectroscopic analysis

#### (a) FTIR study

**Fig. 4:**
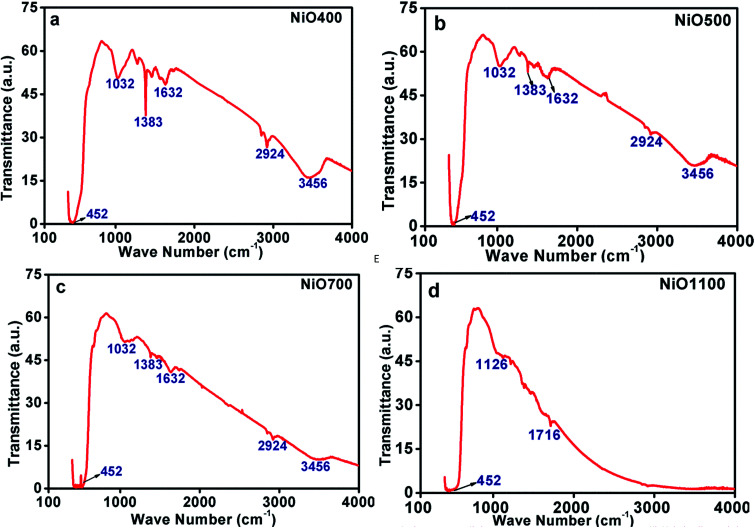
FTIR of non-stoichiometric Ni_1−*δ*_O samples for (a) NiO400, (b) NiO500, (c) NiO700 and (d) NiO1100.

FTIR spectroscopic study of Ni_1−*δ*_O samples provides valuable information about the phase composition and the way in which oxygen is bonded to metal ions. Fig. 4(a–d) show infrared (IR) transmission spectrums of NiO400, NiO500, NiO700 and NiO1100 samples and Fig. S3(a–d) in ESI† shows NiO600, NiO800, NiO900 and NiO1000 having different oxygen contents and oxidation state in the range between 400 and 4000 cm^−1^. The spectrum exhibits a noticeable shoulder peak in the region of 440–460 cm^−1^. For NiO700 to NiO1100 samples, this peak attains a maximum at nearly 460 cm^−1^ and a slight shifting of 10 to 20 cm^−1^ for non-stoichiometric NiO400 to NiO600 samples is noticed. The observation of such peak in the long wavelength region, analogous to previous reports,^27^ could be assigned to the Ni–O stretching vibration mode and the shifting is an indication of the non-stoichiometry present in these samples. In fact, in this long wavelength transverse optical mode, in which the sublattice of Ni^2+^ ions moves 180° opposite to the sublattice of O^2−^ ions for bulk NiO has been reported to lie between 390 and 405 cm^−1^.^28^

There exist other intensive peaks at 1032, 1383 and 1612 cm^−1^ in Ni_1−*δ*_O samples prepared below 700 °C. The existence of these bands clearly indicates the existence of organic molecules such as nitrate ions, water molecules and/or hydroxide ions. It is instructive to mention that the thermal analysis evidently identifies that the Ni(NO_3_)_2_·6H_2_O was decomposed completely to NiO at temperatures higher than 600 °C.^24^ Hence, at the decomposition temperature up to 600 °C, such organic molecules remains in samples leading to the evolution of transmission bands as mentioned above. Further, some of the bands are found to be disappeared as the decomposition temperature increases as TGA data indicts that at higher sintering temperature excess oxygen decreases.^24^ The peaks in IR spectrum at 2924 and 3456 cm^−1^ could be assigning to the presence of carbon in these samples.

These FTIR data (Fig. 4(a–d)) are thus consistent with those found in TGA (Fig. 3(a–d)) and iodometric analysis data. Therefore, using the vacancy model, we can explain the variation of the hole concentration of nickel oxide. Ni_1−*δ*_O is a typically metal-deficient metal oxide. Nickel vacancies are formed at nickel cation sites in NiO due to excess of oxygen ions. The nickel vacancies created at the cation sites can be ionized to create Ni^3+^ ions *via* the following reaction^29^1
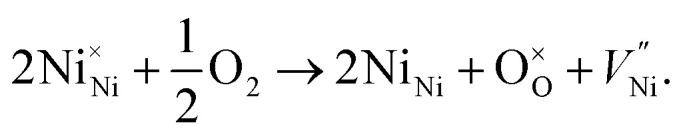


Two Ni^2+^ ions 
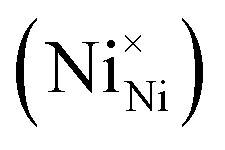
 which react with oxygen produce one ionized nickel vacancy 
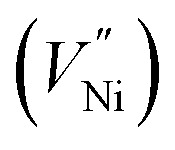
 and two Ni^3+^ ions 
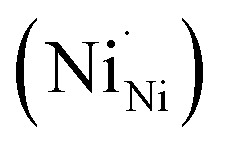
 in the NiO crystal. Each created Ni^3+^ ion serves as donor that donate a hole and also affect the conductive property of nickel oxide. The results of iodometric titration, TGA and FTIR analyses demonstrate that non-stoichiometric nickel oxide is nickel-deficient. Here, according to eqn (1), we argue that an increase in number of nickel vacancies created can induce an increase in the number of Ni^3+^ ions, which in turn increases the hole concentration of non-stoichiometric Ni_1−*δ*_O and it becomes a p-type semiconductor.

In fact, the majority of defects in Ni_1−*δ*_O inferred to cationic vacancies compensated by electron holes. This model has been confirmed by a Seebeck coefficient,^30^ electrical conductivity^31^ measurements as well as kinetic measurements of the rate of oxidation of nickel metal to nickel oxide.^32^ This defect processes play a vital role in determining the properties of metal deficit nickel oxides and is strongly influenced by the extent of excess oxygen present in the polycrystalline sample. The atomistic simulations can be done to calculate inter-atomic potentials and a sufficiently large inner region, these methods can produce accurate values of the energies of defect formation, migration, and substitution.^33^ In the present case, the potential describing interionic interactions is represented by ionic pair-wise potentials of the form2
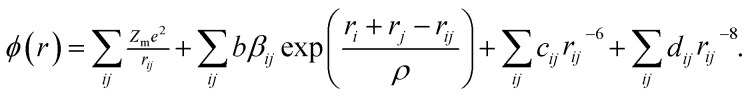
here, the first term represents long-range Coulomb, the second term corresponds to Hafemeister and Flygare form of short-range repulsive energies^34^ and van der Waals multipole are represented by third and fourth terms, respectively. The symbols: *c*_*ij*_ and *d*_*ij*_ are the van der-Waals coefficients and *β*_*ij*_ is the Pauling coefficient, respectively. *Z*_m_ is the modified ionic charge and parametrically includes the Coulomb screening effect, while *b* and *r* are short-range parameters. Thus, the effective interionic potential contains only three free parameters (*Z*_m_, *b*, and *r*), which can be determined from the crystal properties.^35^

The short-range potential parameters assigned to each ion–ion interaction were derived by empirical fitting to observed structural properties. In the context of the columbic term, integral ionic charges are presumed, *i.e.*, 2^+^ for Ni and 2^−^ for O, which enables a straight forward definition of hole states as Ni^3+^ or O^−^. The deduced potential parameters are listed in Table 3 for all the samples. It is clear from the calculated parameters as the sintering temperature of precursor increases the nature of ordering in systems in which ions occupy sites on a face-centered-cubic (fcc) lattice changes the nearest-neighbor (NN) and next-nearest neighbor (NNN) exchange interactions. This happens in such a manner that the structure of NiO undergoes a week cubic-to-rhombohedral distortion as a result of the magnetostriction effect in the presence of excess oxygen in the samples.^36^ The NN and NNN exchange interactions and the antiferromagnetic (AFM) structure of NiO are altered due to the presence of excess oxygen in the samples. Since the radius of Ni^3+^ ions is smaller than that of Ni^2+^ and hence the Ni^3+^–O^2−^ bond contributes to the shorter bond distance in those samples whose oxygen content is higher. In due course, interatomic potential parameters and bond length change as the sintering temperature changes, the magnetic ordering transition temperature is expected to change as we will discuss in later sections.

**Table 3:** Interatomic potential parameter of nickel oxide sintered at different temperatures as discussed in the eqn (2)

Samples	*b* (10^−12^ erg)	*Ρ* (Å)	*ϕ* (eV)
NiO400	39.51	0.388	−39.592
NiO500	39.46	0.388	−39.602
NiO600	39.40	0.388	−39.612
NiO700	39.38	0.388	−39.623
NiO800	39.40	0.388	−39.612
NiO900	39.41	0.388	−39.612
NiO1000	39.57	0.388	−39.518
NiO1100	39.41	0.388	−39.612

#### (b) XPS study

**Fig. 5:**
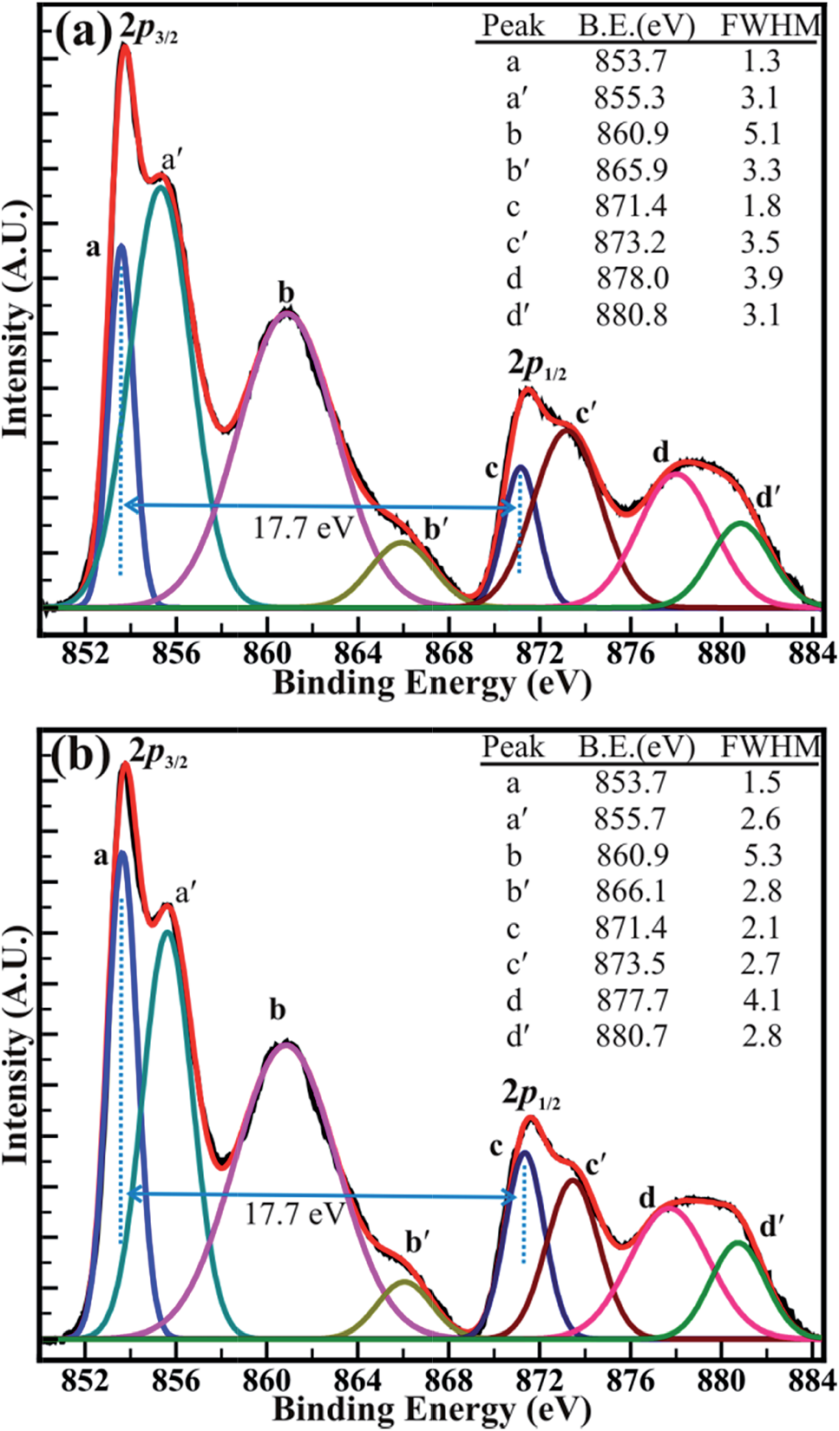
High-resolution XPS spectra of the Ni (2p) core levels of the Ni-oxides decomposed at a temperature of (a) 500 °C and (b) 700 °C. The XPS spectra were decomposed using Voigt peak function fittings.

XPS studies were performed to investigate the stoichiometry and chemical properties of nickel oxide. Fig. 5(a and b) shows the high-resolution XPS spectra of Ni (2p) core level recorded for the NiO samples decomposed at a temperature of 500 and 700 °C. The high-resolution XPS spectra of the samples decomposed at a temperature of 400 and 1100 °C are provided in ESI (Fig. S4 in ESI†). All observed XPS spectra illustrated distinct five peaks located at various binding energies. However, double peak features representing the Ni (2p) core levels for NiO were observed in all samples. For precise determination of the double peak features of Ni (2p_3/2_) and Ni (2p_1/2_), the XPS spectra were decomposed using Voigt peak fitting function within the Shirley background. The perfect fit for 8 peaks marked as a, a′, b, b′, c, c′, d, and d′ are located at binding energy of 853.7 (±0.2), 855.5 (±0.2), 860.6 (±0.2), 865.9 (±0.2), 871.4 (±0.2), 873.3 (±0.2), 877.9 (±0.2), and 880.7 (±0.2) eV, respectively. The peaks marked as a, a′, c and c′, represent core levels of Ni^2+^ (2p_3/2_), Ni^3+^ (2p_3/2_), Ni^2+^ (2p_1/2_), and Ni^3+^ (2p_1/2_), respectively. The decomposed shake-up satellite peaks (marked as b, b′, d and d′) were observed at ∼7.1(±0.2) or 10.2(±0.2) eV and ∼6.3(±0.3) or ∼7.1(±0.2) eV higher in binding energy than that of Ni^2+^ (2p_3/2_), Ni^3+^ (2p_3/2_), Ni^2+^ (2p_1/2_), and Ni^3+^ (2p_1/2_) peaks, respectively. The appearance of double peak features of Ni (2p) along with their consecutive shake-up satellite peaks are indicative of the magnetic chemical state of Ni^2+^ and Ni^3+^ state.^37^ These observations confirm that the peak positions are akin to those reported in previous studies for the presence of Ni^2+^ and Ni^3+^ oxidation states in stoichiometric and non-stoichiometric NiO.^38^ Likewise, the representative O (1s) XPS spectra of the sample decomposed at a temperature of 1100 °C shown in Fig. S5 in ESI† was decomposed using Voigt peak fitting function within the Shirley background. The oxygen spectra show a perfect fit for two peaks located at a binding energy of 529.3 and 531.1 eV with FWHM of 1.2 and 1.7 eV, respectively. The lower binding energy peak observed at 529.3 eV corresponds to the O (1s) core level of O^2−^ anions associated with Ni–O chemical bonding. However higher binding energy peak observed at 531.1 represents the surface contamination or presence of hydroxyl (–OH) groups.^38–41^

**Fig. 6:**
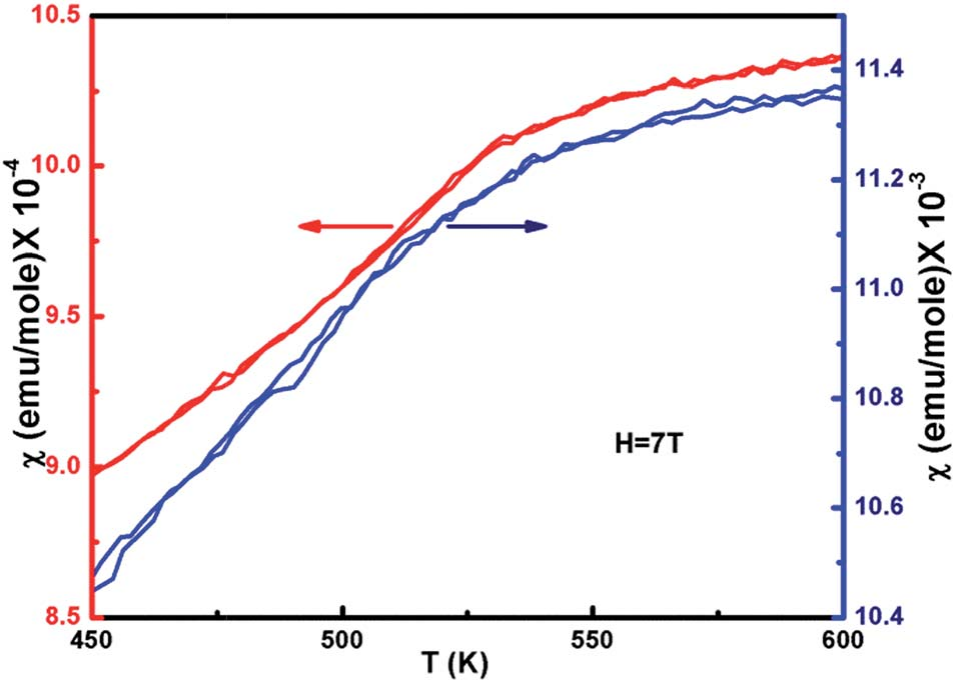
Magnetization for ZFC and FC curves of non-stoichiometric Ni_1−*δ*_O samples for NiO400 and NiO1100 in 7 T applied field as a function of temperature.

The close analysis of decomposed XPS spectrum revealed that the intensity of the peaks assigned to core levels of Ni^3+^ (2p_3/2_) and Ni^3+^ (2p_1/2_) is larger than that of core levels Ni^2+^ (2p_3/2_) and Ni^2+^ (2p_1/2_) at decomposition temperature of 400 and 500 °C. However, the intensity of the peaks of Ni^2+^ (2p_3/2_) and Ni^2+^ (2p_1/2_) core levels has increased than that of peaks of Ni^3+^ (2p_3/2_) and Ni^3+^ (2p_1/2_) core levels after the decomposition temperature of 700 °C and are continued to increase for the temperature of 1100 °C. The intensity ratio obtained for the peaks of the Ni^2+^ (2p_3/2_) and Ni^3+^ (2p_3/2_) core levels (*i.e.* Ni^2+^/Ni^3+^) has increased from 0.85 (±0.03) to 1.23 (±0.03) with an increase in the temperature from 400 to 1100 °C. Moreover, the variation in the intensity of the core levels assigned to the Ni^2+^ and Ni^3+^ is more apparent at decomposition temperature of 500 and 700 °C in Fig. 6(a and b). This can be assigned to the transformation of Ni^3+^ ions into Ni^2+^ ions with increasing temperature. Furthermore, the binding energy difference (Δ*E*) of 17.7 (±0.1) eV between the Ni (2p_3/2_) and Ni (2p_1/2_) peaks is very close to that of 17.8 eV for oxidized Ni and significantly larger than that of 17.2 eV for metallic Ni.^42^ This confirms again that the ‘Ni’ is materialized in its oxidize forms (*i.e.* Ni^2+^, and Ni^3+^) and not in its pure metallic form. Overall, XPS investigation confirms that the non-stoichiometric NiO comprising a higher proportion of Ni^3+^ ions at 400 °C has significantly transformed into stoichiometric NiO at 1100 °C, which is composed of more Ni^2+^ ions. This analysis from the XPS spectrum shown in Fig. 5(a and b) and S4(a and b)† supports our TGA analysis reported above in this manuscript. Therefore, it can be concluded that the sintering temperature can assist to control the relative concentration of the nickel–oxygen species, distinctly. By controlling the sintering temperature non-stoichiometric nickel oxide can be prepared.

### 3.4 Magnetic properties measurements

The temperature dependence of the magnetic susceptibility (*χ*) for selected samples of NiO400 and NiO1100 is plotted in Fig. 6 with an applied magnetic field of 7 tesla. For different stoichiometry, the magnetic susceptibility (*χ*) values were measured for both the zero-field-cooled (ZFC) and field-cooled (FC) conditions and a reversible behavior with negligible hysteresis of *χ* for different stoichiometry was evident. Both samples have paramagnetic (PM) to antiferromagnetic (AFM) transition at the Néel temperature *T*_N_ (defined as the slope change in the *χ vs. T* curve) and the measured values are 480 and 530 K for NiO400 and NiO1100 samples, respectively. It can be seen from the figure that both the *T*_N_ and the absolute value of *χ* for NiO400 sample are lower than that of NiO1100 sample. However, PM to AFM transition width appears to be broader for the NiO1100 sample. We argue that the enhancement in *T*_N_ with stoichiometry could be attributed to the effect of the partial destruction of Ni^2+^–O–Ni^3+^ exchange interaction network because of the reduction of the oxygen vacancies and the weakening of Ni^2+^–O–Ni^3+^ interaction arising from the decrease of the bandwidth of e_g_ electrons due to the change in Ni–O bond length and Ni–O–Ni bond angle.^43^

### 3.5 Specific heat measurement

**Fig. 7:**
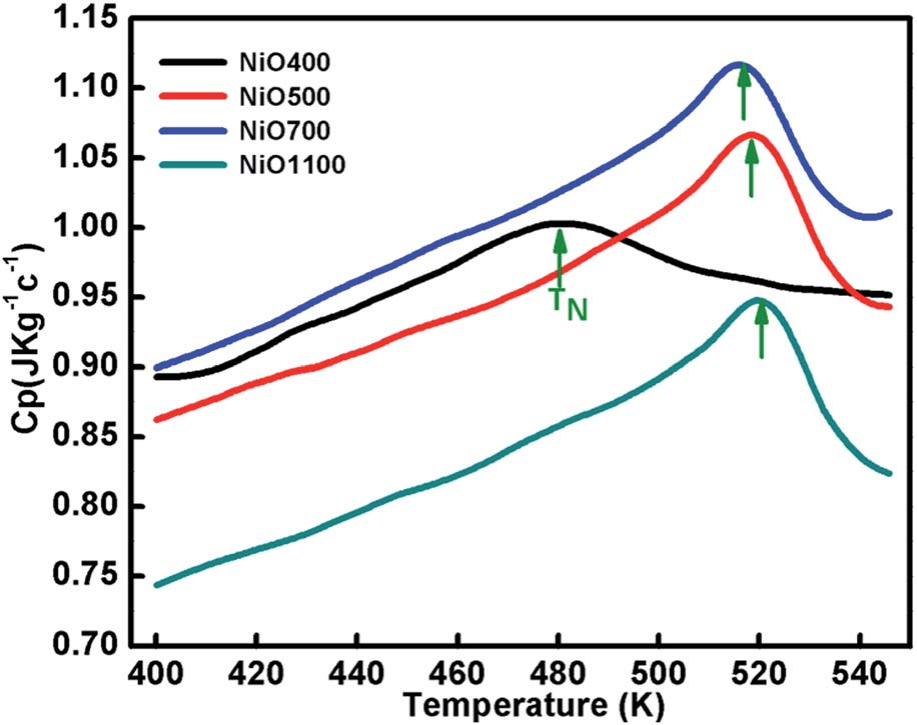
The temperature variation of specific capacity of nickel oxide samples sintered at different temperatures. Curve shows shifting in *T*_N_ due different oxygen contents of the samples.

Specific heat measurements (*C*_p_) for various samples of nickel oxide for different stoichiometry are presented in Fig. 7. The specific heat anomaly is evident in the vicinity of *T*_N_, a clear indication of the AFM ordering in these samples. As the stoichiometry of sample changes, a significant change in the anomaly is observed. Further, a gradual increase in transition temperature from NiO400 to NiO1100 samples is evident as the sintering temperature increases. The obtained values of transition temperature are 510, 519 and 525 K for NiO500, NiO700 and NiO1100 samples, respectively. It can be seen that due to the presence of excess oxygen in the different ratio in NiO400 and NiO500 samples, change in specific heat anomaly is observed. Further, as the excess oxygen almost disappears in the high temperature sintered samples, the observed transition temperature reaches 525 K as reported for stoichiometric NiO.^44^ Such an anomalous behavior in *C*_p_ at *T*_N_ is due to Ni-spin ordering as suggested by Néel,^45^ wherein the spin–lattice of the particle could reverse coherently and randomly under thermal activation. Consequently, the net moment of uncompensated surface spins would fluctuate accordingly and in turn, significant downshift the *T*_N_ in samples due to both the change of magnetic ions as well as their disorder. Presumably, a small amount of excess oxygen could increase magnetic inhomogeneity in samples and eventually shift the Néel temperatures.

## 4. Conclusions

Nickel oxide samples of different stoichiometry were prepared by thermal route method and samples were thoroughly characterized by XRD indexed by full-proof refinement. Their non-stoichiometry was established by iodometric titration and TGA, and the excess oxygen of samples was estimated. The FTIR studies indicated the presence of NiO phase with some amount of hydration and nitrate ions. It also confirms the non-stoichiometry of samples. XPS results revealed that nickel vacancy can be created in samples with varying sintering temperatures. Besides, an excess of Ni^3+^ ions was noticed in samples sintered at lower temperatures. The temperature variation of *χ* for ZFC and FC curves is nearly identical to each sample, while the magnitudes of *χ* differ for Ni_1−*δ*_O samples with different values of *x*. The observed specific heat anomaly in the vicinity of *T*_N_ is associated with the magnetic ordering, indicating a gradual transformation between two magnetic phases and the observed *T*_N_ shifted towards lower temperatures as excess oxygen content increases. The shifting of Néel temperature is presumably due to magnetic inhomogeneity arising from the excess oxygen in samples.

## Conflicts of interest

There are no conflicts to declare.

## Supplementary Material

RA-008-C8RA00157J-s001
