# Supplementary material for: The effect of stoichiometry on the structural, thermal and electronic properties of thermally decomposed nickel oxide
Source: RSC Adv. 2018 Feb 6;8(11):5882–90. doi: 10.1039/c8ra00157j (PMC9078242; doi:10.1039/c8ra00157j)
Supplement: RA-008-C8RA00157J-s001 [file RA-008-C8RA00157J-s001.pdf]

Electronic Supplementary Material (ESI) for RSC Advances.
This journal is © The Royal Society of Chemistry 2018

The effect of stoichiometry on the structural, thermal and electronic properties of thermally decomposed nickel oxide

P. Dubey,[a] Netram Kaurav,[a,*] Rupesh S. Dewan,[b] G. S. Okram[c] and Y. K. Kuo[d]

[a]Department of Physics, Government Holkar Science College, A. B. Road, Indore 452001, MP, India.
[b]Discipline of Metallurgy Engineering and Materials Science (MEMS), Indian Institute of Technology Indore, (IITI), Khandwa Road, Simrol, Indore 453552,MP, India.
[c]UGC-DAE Consortium for Scientific Research, University Campus, Khandwa Road, Indore 452001, MP, India.
[d]Department of Physics, National Dong Hwa University, Hualien 97401, Taiwan

**Corresponding Author email: netramkaurav@yahoo.co.uk*

Supplementary Materials:

Figures S1 to S5

1. **XRD data:** Fig. S1 shows X-ray diffraction pattern of non-stiochiometric $Ni_{1-\delta}O$ samples sintered at different temperature as indicated. The cell parameters of all samples listed in Table 1.

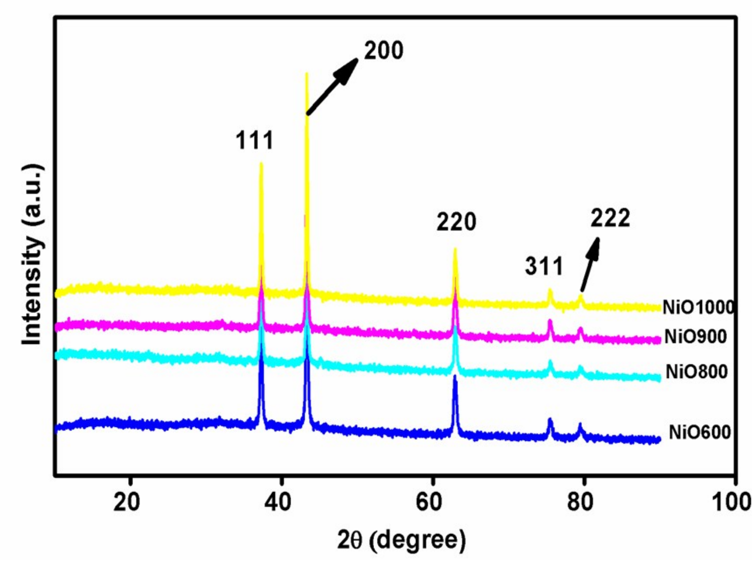

Fig.S1 X-ray diffraction pattern of non-stiochiometric $Ni_{1-\delta}O$ samples sintered at different temperature as indicated.

2. TGA data: Fig. S2 shows TGA curves of NiO400 sintered in presence of oxygen with heating rate of 5°C per min was maintained. The excess oxygen calculated is nearly equal to the sampled prepared normally in presence of air.

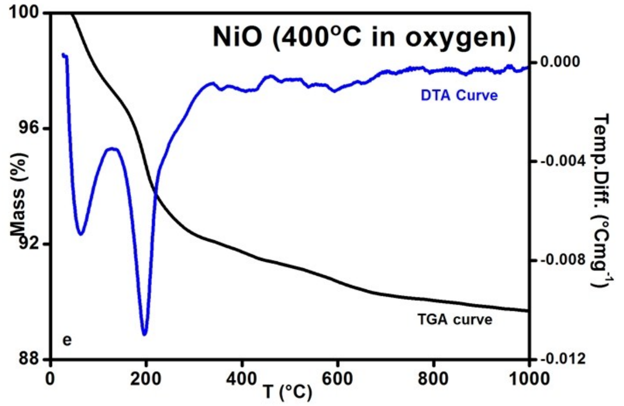

Fig. S2 TGA curves of NiO400 sintered in presence of oxygen with heating rate of 5°C per min was maintained. The excess oxygen calculated is nearly equal to the sampled prepared normally in presence of air.

3. **FTIR data:** Fig. S3 (a-d) shows the FTIR of non-stoichiometric Ni1-δO samples

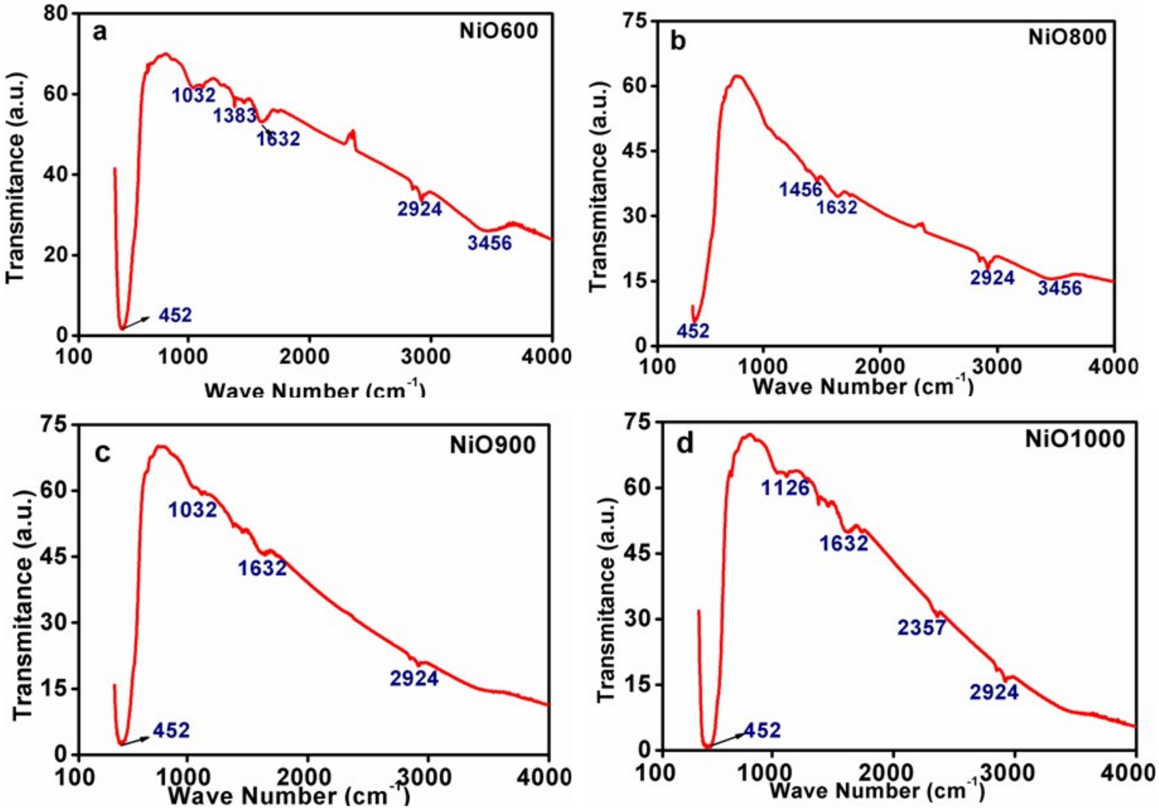

Fig. S3 (a-d) FTIR of non-stoichiometric $Ni_{1-\delta}O$ samples for (a) NiO600, (b) NiO800, (c) NiO900 and (d) NiO1000. These figures indicates the change in stoichiometry as the sintering temperature changes.

4. XPS data

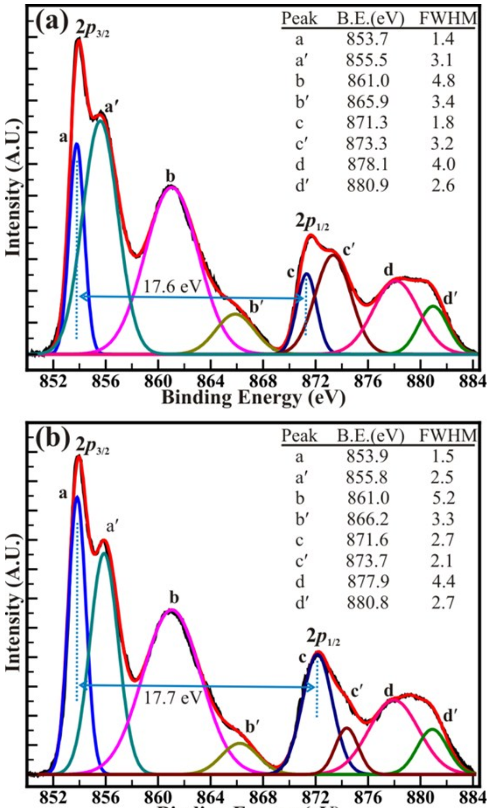

5.

Fig. S4 High-resolution XPS spectra of the Ni-oxides decomposed at a temperature of (a) 400 ^{o}C and (b) 1100 ^{o}C. The XPS spectra were decomposed using Voigt peak function fittings.

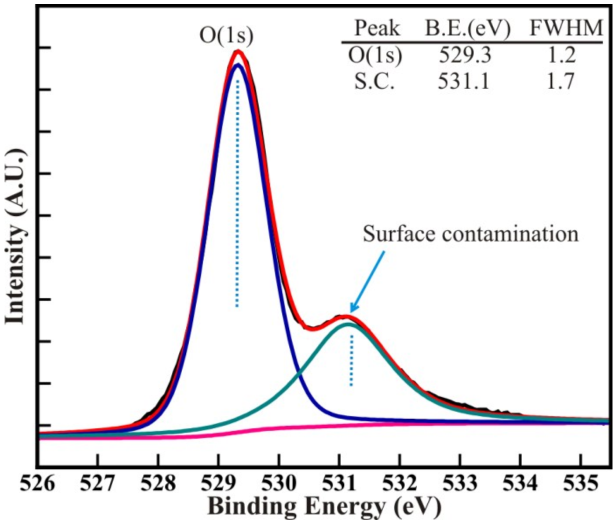

Fig. S5 High-resolution XPS spectra of the O(1s) core level of the Ni-oxides decomposed at a temperature of 1100 ^{o}C. The XPS spectra were decomposed using Voigt peak function fittings.
